# A digital humanism view on e-tourism

**DOI:** 10.1007/s40558-022-00237-6

**Published:** 2022-10-11

**Authors:** Hannes Werthner

**Affiliations:** E-Commerce Research Unit, TU Wien, Vienna, Austria

**Keywords:** Digital Humanism, e-tourism, Digital transformation, Downsides of IT, Resilience

## Abstract

Informatics, its artefacts and methods, change our society and world, from the individual personal level up to the current geo-political powerplay. Today, Information Technology (IT) serves as the operating system of our society. From an ontological point of view Informatics influences also how we perceive the world and how we think about it. This change has happened during the short span of last half century. At the same time, despite of this enormous success, this development has serious shortcomings. I discuss some of them, and describe a positive “answer”: Digital Humanism. I will use this human centered approach to IT to critically review developments in e-tourism; and, one of the main lessons is: do not get lost in small things, have a look at the big picture, also aim for a better future and society. Two disclaimers: (a) This contribution is an opinion piece, not a scientific paper in the strict sense. I hope it provokes a lively, and probably also controversial discussion; and, (b) Some arguments regarding Digital Humanism are already partly expressed in other publications such as (Werthner, Electron Mark 32:145–151, 2022a) and (Werthner et al. Hoepken (eds) Handbook of e-tourism, Springer, Berlin, 2022b).

## Introduction

“This is absolute nonsense” was the response by the audience, I remember, at the first ENTER conference on IT and tourism in Innsbruck in 1994. Beat Schmid (University of St. Gallen) spoke about electronic markets; and Larry Press (University of California, Los Angeles) foresaw digital agents as copies of ourselves in the digital world. The majority of the audience—both scientific as well as industrial—was really skeptical.^1^

Today we experience, being also astonished by its transformative power, the complex, technical socio-economic process called *Digital Transformation*, which was called nonsense only 35 years ago. This transformation from a “stand-alone” computer to a worldwide mega-machine touches on every aspect of our lives. IT acts as the “Operating System” of our society. As stated by Lee (2020), we experience the co-evolution of man–machine. But this development has also its downsides, with negative impact in the economic, societal and political arenas.

This can also be observed in tourism, as this industry has even been one of the first and important application fields of IT already back in the 60 s. In this viewpoint I will have a critical look at this IT-based and -induced changes, which transformed the industry. I will describe some of these critical issues and offer a positive answer called *Digital Humanism*. To understand this development, one also has to look at Informatics, the Web and the platform economy. In the final part I will provide a short and subjective review of the e-tourism development, and then use the Vienna Manifesto on Digital Humanism (Werthner 2020) to critically reflect on this development.

## “The system is failing”

IT systems are useful and extremely successful. When looking at the COVID-19 pandemic as an example, without “our” tools the world would have stopped” including our work, our school, our personal and public communications, and also the research for proper vaccines. It is hard to think of all that without IT. It kept and keeps the system running, and it serves for solving fundamental and vital problems (e.g., the essential role of informatics to tackle the Sustainable Development Goals (SDG) of the UNO^2^). At the same time, this development comes with downsides, as stated already in 2018 by Tim Berners-Lee: “The system is failing”.^3^ The list of critical and mutually dependent issues is long:

- Concentration and monopolies in the Web, where multinational technology companies have power that national democratic governments have serious problems to control. These companies offer services states do not provide with that quality, they decide—and not the states—on the implementations of crucial services for citizens.
- The role of IT has become so essential on economic, political and even military level that it is a critical issue of geo-political sovereignty.
- AI and automated decision-making—put simply, the representation and automation of human thought—may result in autonomous decision-making systems, with substantial legal and ethical questions (Larus et al. 2018). What makes it worse, in many cases (when AI is based on black-box algorithms), we do not understand the outcome, i.e., decisions proposed and taken.
- Further automation will have massive impact on employment and jobs—in both qualitative and quantitative terms. How are and will these “new” jobs be designed? What Is the role of the human in the loop? And does not the IT industry reproduce post-colonial division of labor as described by Casilli (2021)?^4^^,^^5^
- We see violations of privacy on a massive scale, both by private companies and by state instances, well described by Zuboff (2019).
- These developments are also evident in the political arena, e.g., in the intentional fabrication of fake news and the creation of opinion bubbles in the Web.
- AI moves to warfare, resulting into autonomous weapons. Already U.N. Secretary-General António Guterres states that “Autonomous machines with the power to …. take lives without human involvement…. should be prohibited by international law.”^6^

## Digital humanism and its Vienna manifesto

This IT “double face” was the motivation for the *Digital Humanism Initiative*, with a first workshop in 2019 in Vienna. Over 100 attendees from academia, government, industry, and civil society participated in this lively two-day workshop. We talked about technical, political, economic, societal, and legal issues, benefiting from contributions from different disciplines such as political science, law, sociology, history, anthropology, philosophy, economics and informatics. At the center of the discussion was the relationship between informatics and society, or, as expressed during the workshop, the co-evolution of information technology and humankind. The major outcome was the *Vienna Manifesto on Digital Humanism*, now available in eight languages, which lays down the core principles of our initiative.

The term *Digital Humanism* was intentionally chosen to refer to the concepts of Humanism and Enlightenment, according to which the human is responsible for his or her own thinking and is at the focus (Nida-Rümelin and Weidenfeld 2018). We also underline the importance of rational and critical reasoning, which is a reference to the Vienna circle and its logical empiricism (Sigmund 2017). We have the freedom, the right and the responsibility to make use of our own thought power, we are the authors of our own lives; personal autonomy and freedom to make decisions are the prerequisites for an open, democratic society. Technological progress is not God-given. We as individuals and as society should and must make decisions taking democratic and humanistic considerations into account. We define *Digital Humanism as an approach that describes, analyzes, and, most importantly, influences the complex interplay of technology and humankind, for a better society and life, fully respecting universal human rights*.

The term *humanism* refers to two rather different movements. The first denotes the period between mid-15th until end of sixteenth century (Renaissance Humanism), with a rediscovery of antiquity in the arts and in philosophy. Aesthetics and ethics became centered on humans, rather than on the supernatural or divine. A second period of humanism flourished in the Enlightenment period (end of eighteenth century), and the French revolution was largely inspired by the principles of human freedom and democracy rooted in the humanistic spirit of that time. Naturally, the two movements share a range of common concepts and interests, some of which remain relevant for Digital Humanism today, for example, a strong focus on human rights and how to maintain them in the digital realm.^7^

The workshop’s *Vienna Manifesto for Digital Humanism* proclaims the following *principles*^8^:

Privacy, democracy and inclusion

- Digital technologies should be designed and deployed in such a way that they promote democracy and inclusion.
- Privacy and freedom of speech are basic values which should be at the center of our activities.

Regulation and public oversight

- The regulatory authorities must intervene to break up technology monopolies.
- Decisions whose consequences could affect individual or collective human rights must still be made by humans.

Specific role of science and the academic sector

- Scientific approaches integrating various disciplines and eliminating discipline-specific silos are needed for mastering our challenges.
- Universities are the places where new knowledge is created and critical thinking is exercised. They should eliminate the boundaries among disciplines and fostering their collaboration toward a holistic view of technological development.

*Education and training*

- New curricula are required which combine Humanities, Social Sciences, and Technical and Engineering Sciences.
- Education in IT and training work on the ethical and societal impacts of IT must begin as early as possible in the education process.

We touched an obvious hot topic; there was a very positive response from academics from different disciplines, from civil society, funding agencies and even political decision makers. There are a number of international initiatives with similar objectives with which we started to network, e.g., HAI—Human-Centered Artificial Intelligence at Stanford, Center for Humane Technology, or the Dutch Digital Society. The growing public awareness is also reflected by several recent political international actions; e.g., in the US antitrust lawsuits against Facebook and Google, in Europe *Digital Service Act, Digital Market Act*, proposal for AI regulation, or the European GDPR, all focusing on a regulation of the online world. Other examples are the OECD’s principles on AI, UNESCO’s activities or the global Partnership on AI. And international standardization organizations moved, e.g., IEEE with its IEEE 7000 Software Engineering Standard.

Due to the pandemic our activities moved online with since then over 30 regular public online lectures and four workshops. with a growing number of participants, internationally renowned speakers and with topics ranging from AI and ethics, COVID-19 apps and privacy, to the issue of sovereignty in the digital world.^9^ In addition, we also published the volume *Perspectives on Digital Humanism* (Werthner et al. 2022) with currently already over 190.000 downloads, formulated a *DigHum Roadmap for Research, Innovation and Teaching* (Prem et al. 2022), and organized a successful Summer school.^10^ But most important, we succeeded in creating an intellectual core and community consisting of many international renowned colleagues from all disciplines involved.

## Some notes on informatics

The basis of this development is the power of the Computer and its science, Informatics.^11^ The computer is a general-purpose automaton, which can, as the only automaton, control itself via software, and be instantiated by software to any particular specific problem-solving machine, e.g., to control powerplants or to play a game. This machine has the unique property of being able to independently change and control its own behavior, based on external inputs and internal states, it shows some form of self-reflectively.^12^

IT became the global operating system^13^ of our society, it integrates, links and permeates everything: work, leisure, politics, the personal, the professional and the private. At the same time, IT becomes invisible and increasingly “disappears”. Current technical and even other systems consist of a stack of different hardware and software pieces (not only computing machines, but also any other machine, be it traffic systems or individual vehicles), with tasks increasingly being delegated to software. This leads to more and more virtualization. Thus: **Everything touched by software becomes a computer!**

In this context I use the broad definition of the Turing Award winner Kristen Nygaard that “Informatics is the science that has as its domain information processes and related phenomena in artifacts, society and nature” (Nygaard 1986). As such Informatics is a fundamental science of today, and its methods influence how we perceive the world and how we think about it. Its artifacts change the world. As such, informatics is also affecting other sciences, either as a tool or as another ontological approach. The importance of Informatics is also revealing its interdisciplinary nature and extensive connections with engineering, technical and social sciences, and humanities: it is versatile in scientific computations and simulations; it has changed the practice in other disciplines. In addition, Informatics creates new things both virtual and real. It is the only (engineering) discipline that creates systems without being limited by physical constraints, which is similar to arts.

## The web

The Web as the currently most influential socio-technical system of our times is an excellent example of the power of Informatics. It is—to a wide extent—an unregulated public resource. However, Hardin (1968) already describes the “Tragedy of the Commons", referring to the phenomenon where individual users acting independently according to their own self-interest behave contrary to the common good—and “destroy” the latter.^14^

Historically, one can link the Internet, and subsequently the Web as the best known application protocol of the Internet, to the US antiestablishment movement and its utopian cultural vision, e.g., the declaration of the independence of the cyberspace (Vardi 2018). The original idea of the freedom of information with information as a free public good and free information sharing led to a huge amount of freely available information, and, consequently, the rise of search engines. The problem, however, was how to sustain or to monetize such a world-wide information system with no clear income business model. The solution was and is an advertising-based business model, similar to newspapers, but extended by *personalization* (see Jeff Bezos’ claim that “If we have 4.5 million customers, we have 4.5 million stores”) and *recommendations*. Both, personalization and recommendation, are essential to “find something” in this exploding information and product universe. They led to changing user behavior in that users adapt and follow recommendations.^15^ As the basic unit of “return” is clicks and one needs to optimize clicks, the Web became emotionalized, where negative emotions generate more clicks. Advertisers pay for user data, leading to the well described surveillance capitalism (Zuboff 2019), we—users—became consumers instead of citizens (Stanger 2020). At the end we are users, products and producers at the same time—nearly an economic perpetuum mobile.

A second observation: In the Web we seem to have absolute individual freedom. But this is a delusion: *what I see*, *what I get*, *what I do* is essentially defined by the distance measure of a recommender’s similarity matrix. In addition, there is an algorithmic interdependence between the individual (reinforced by our almost narcissistic and exuberant self-referentiality, see selfies) and the general/common.^16^ But also, this “common” is a fiction as it is an aggregation of previously individualized views. In essence, instead of the conscious decision of the human for what to see and what to do, this is done by a mostly unknown algorithm. At the end we have the delusion of both, the individual freedom and the seemingly common. The original vision of individual freedom and democratic participation led to a failing systems, as expressed by Berners-Lee. In this context, I see Digital Humanism as a rational democratic answer to the nearly postmodern “anything goes” paradigm of the Web. In this view it is also a socio-cultural issue, not only a technical one.

Online platforms

The Web lays the foundation of a further important development, i.e., platforms and platform economy. These new companies have a self-understanding as IT companies, are coming from outside, and diffusing into all economic and societal sectors with new technical and market services. Platforms are currently the dominant online intermediation structure and economic environment. Based on a common architecture and a set of transactional rules these platforms could also be characterized by a kind of dialectic relationship between cooperating networks and at the same time centralization around platform operators, which create and control these structures. Its network effects with its dynamics and the *winners take it all* phenomena led to a situation where a small number of players dominate the market (Parker et al. 2016; Parker 2019). They increase market efficiency through the reduction of transaction costs (Williamson 1985). From on organizational perspective platforms can be viewed as a new form of an organizational model, in addition to hierarchies, markets and networks (Stark and Pais 2021). They show similarities to all three forms, but cannot be reduced to one of them.

In June 2022, when looking at market valuation, six of the top 10 companies were platform companies, showing their economic power. Compare this with the year 2013 when only two of those companies were in the top 10.^17^ In some markets these platforms have markets shares of 50–90 percent. As discussed by (Cusumano^2019^) these companies make their turnover, and approx. double profit, with only half of the number of employees, compared to similar traditional companies.

Focusing on transactional services the big platforms are industrial “sector” independent and, as such, they are orthogonal to industrial domains (in that you can buy “everything” on Amazon), and concrete products play an almost negligible role; they are virtualized, as are companies, entire markets and increasingly our society. Not to forget: these platforms are innovation drivers (Gawer and Cusumano 2013) with platform competition instead of product competition.

At the end we are confronted with a situation where these IT platforms offer services the public cannot do; these services are already public social goods; and, individuals and companies have to participate as not to be excluded from public life. And given the plethora of information, we again need Informatics and “its” intelligent tools to help us navigate the information space (Baeza Yates and Fayyad 2022).

## e-tourism^18^

At the beginning of the Web back in the 1990s, travel and tourism was already one of its major application fields. But even since its early diffusion, IT has played an important role in tourism; in the 1960s Computerized Reservation Systems/Global Distribution Systems (CRS/GDS) were one of the first world-wide electronic networks, laying the basis for the automation of the (air) travel industry with its massive efficiency gains, leading to mass tourism. Also, in this case computing power and technical competence facilitated new market structures and dominant market positions of few players. However, in contrast to the current situation, these systems were industry domain dependent, and, maybe more important, they were created by “traditional” market players.

Looking at the current situation, the HOTREC's distribution^19^ study confirms the dominant role of platforms also in tourism: between 2013 and 2021 the market shares of OTAs (Online Travel Agents as the prototypical new players in the field) have steadily increased in the European hotel sector from 19.7% in 2013 to 29.2% in 2019. At the same time, the share of direct bookings (i.e., direct contact and transaction between a tourist and a hotel) has decreased across Europe from 57.6% in 2013 to 47% in 2019, with a very slight increase during the pandemic. In the OTAs segment the three main players have a share of over 90%, with one player (i.e., booking.com) dominating with a steadily growing share of 71.2%. Here, *the winners take it all* phenomenon also holds. And, self-organization on the side of destinations in the form of destination systems (these were among the first movers on the Web online market, e.g. the Tyrolean system TIScover) remains low with a share of 1–2% of bookings. Interestingly, there is a growing technology sophistication on the supply side with an increased use of channel managers and meta search engines. Obviously, providers help themselves on an individual level, but there seems to be little or no coordination on a joint local/regional level.

As foreseen already in 1994, IT has radically changed the tourism industry; one might even say, IT created a new one. We have a well-developed e-business landscape with new players and structures, and with the “informatization” of the entire value chain. Today, one could state that nearly all companies are “online” companies. As in other sectors, innovation was driven by IT-based newcomers with a fast imitation of business models and technology. Traditional market players had and have problems with service innovation, business models and technology development which seems to have intensified due to the pandemic. The increased competition was accompanied by a focus on efficiency,^20^ i.e., companies optimizing their short-term performance.

What can we expect^21^: With Internet of Things (IoT) the Internet will be everywhere at any time, and it will be “disappearing”, embedded into a plethora of very different devices. On a market structure level, we will see even more concentration. As markets and businesses become even more virtualized, these big platform players will be—based on their IT capacities—able to easily switch between the different markets and market segments. The struggle will be for the ownership of data in order to understand markets, users/consumers and partners/competitors; with a clear advantage for the IT based “newcomers”. They know how to deal with data and how to transform them into knowledge and money.^22^

Back to the present again: Currently, we see a rather “linear” recovery of the pandemic crisis, the business seems to continue as usual. This is opposite to all the public claims that after a crisis we should focus on sustainability, creating a “new” and more resistant and environmentally friendly industry.

## E-tourism and digital humanism

In this final part, I discuss some principles of the Vienna Manifesto in the context of e-tourism. I use the Manifesto—its call to put the human at the center of IT based developments—to critically reflect on current developments in e-tourism and related research. I will conclude with some remarks on efficiency vs resilience as I think that this will be an important issue in the future.

Ad privacy, democracy and inclusion.

- *Digital technologies should be designed and implemented in such a way that they promote inclusion*: this refers to user and stakeholder driven applications, and in the context of e-tourism a reference to destination systems as a “fair” and inclusive representation of regional / local suppliers. However, these destination “platform centric” approaches failed (see also Calatrava et al. 2015), and there seems to be no discussion in this regard anymore. I also miss—at least to my knowledge—tourism specific participatory and human-in-the-loop approaches, as discussed in other fields.
- *Privacy and ownership of data*: this discussion has not yet reached the e-tourism community to its full technical and legal extent and depth. For example, the recommender community discusses the separation of data from algorithm (see Burke and his presentation at the roadmap workshop of digital humanism^23^). I expect privacy to become one of the hottest issues. We should not understand data just as a resource provided by social media to analyze customer satisfaction and then to plan better marketing campaigns. We need to understand biases in tourism data, or how data are misused, or consumers misled.

Regulation and public oversight.

- *The regulatory authorities must intervene to break up technology monopolies.* There was a long discussion on that issue in tourism, starting with the market power of tour operators, and then repeated with respect to the power of the new intermediaries. In the “general” online world this discussion had already some consequences (see the different European regulation approaches). Also, new service specific companies in tourism such as Uber or Airbnb provoked reactions from the public sector as well as private competitors. Interestingly, the industry as well the academic community seems to have accepted the dominant role of OTAs, which is, in my view, structurally more important.
- *Decisions whose consequences could affect human rights must still be made by humans:* there seems to be a rather uncritical uptake of AI in tourism, with very limited discussion of the drawbacks of neural network inherent black box approaches. The focus is rather on adoption issues. This is in strong contrast to other areas and also politics, for example, the EU proposal to regulate AI or the ongoing self-regulation activities of platform companies such as Microsoft.^24^ Research should ask which type of AI applications make sense, which one have the potential to create harm, how AI applications should be designed and implemented.

Specific role of science and the academic sector.

- *Scientific approaches integrating various disciplines and eliminating discipline-specific silos are needed for mastering our challenges*: here e-tourism could play a pivotal role, being interdisciplinary by nature with roots in informatics, sociology, geography, management science, or economics. And it has the experience—more than other disciplines—with life-wide and life-long learning. But we should not focus on professional / technical skills only, but also on critical thinking as one of the core tasks of academia and universities.

The spirit of Digital Humanism is to use digital technologies for a better and fairer world, for the individual and the society. In e-tourism, historically digitalization also had such a flavor, at least in our academic self-understanding. In the following I list—with some self-criticism—several of these “positive” historic expectations, and confront them with the current reality:

- Innovation with new market structures, disintermediation, seamless market participation, and direct contact between supply and demand—> 25new, even stronger intermediaries with more concentration and harder restrictions for suppliers.
- Communities, social media, user participation and empowerment^26^—> user data mainly used for statistical analysis and forecast of user behavior, focus is on better marketing and not on user empowerment
- Sharing economy and direct participation of local community—> exploitation of local communities with new strong intermediaries, and partly abuse (even illegal) of local residential structures, leading to deteriorating housing situation and legal responses by political authorities.
- Social media influencers as a new phenomenon with more direct and honest communication—> new business model for few and mostly used for "hidden" advertising campaigns (many with sponsored followers).
- Blockchain and cryptocurrencies promised direct interaction without central control—> turned out to be in essence a speculation and money laundering instrument, creating massive environmental problems. Also, the discussion on metaverse (and Web 3.0) predominantly looks at new promising business opportunities, but little attention is given on the intentions and interest of the main player (not being known for its world-improving or philanthropic intentions).

As a last issue: In the pandemic there were many loud voices arguing for the need to change tourism, to make it socially and environmentally more sustainable.^27^ In this respect, Digital Humanism may also provide some guidance, since it argues for sustainable (social and ecological) and, consequently, resilient and fault tolerant systems. For example, the digital humanism roadmap for innovation and research highlights the opposing pair efficiency vs. resilience as a major issue (Prem et al. 2022). Resilient architectures having buffers and tolerating faults, and not focusing on optimality, are one of the main lessons, not only from the pandemic (Vardi 2022), but also of the current energy crisis. We need to apply these lessons for the global ecological situation, which is central for tourism as an industry highly dependent on natural resources. We need resilient systems, which have the capacity to adapt to disruptive changes in the environment.^28^

In the past, the logic was to develop and implement efficient systems, which prevented us from investing in getting ready for critical situations. See also Thomas Friedman, in New York Times, May 30, 2020: “Over the past 20 years, we’ve been steadily removing man-made and natural buffers, redundancies, regulations and norms that provide resilience and protection when big systems—be they ecological, geopolitical or financial—get stressed. …. We’ve been recklessly removing these buffers out of an obsession with short-term efficiency and growth, or without thinking at all.”

Economic efficiency means goods and factors of production are distributed or allocated to their most valuable uses, buffers and waste are minimized. Free-market advocates argue that through individual self-interest and freedom of production as well as consumption, economic efficiency is achieved and the best interest of society, as a whole, are fulfilled. One can refer to the *first welfare theorem,* which states that under certain assumptions free markets will produce economic efficiency. However, assumptions such as perfect competition, perfect information or no external costs normally do not hold. Free markets do not guarantee that the best interests of society, as a whole, are fulfilled. Market intervention is needed, as it can be seen in the current situation.

COVID-19 is just a harbinger of a larger challenge: climate change and the ecological crisis. For mastering this (not only in tourism), one needs to look at resilience and at the big picture. However, most cited papers and most discussed topics in e-tourism research are ones on marketing and management related issues such as customer satisfaction, business performance or self-service technology (Molina-Collado et al. 2022).^29^ It seems that research is talking about “micro” issues while losing the big picture. But academia should not forget this perspective as stated in the Vienna Manifesto on Digital Humanism.

## Conclusions

Travel and tourism illustrates well the transformative power of IT. As such, this sector is a prominent example of the overall IT-driven development. IT will not stop, nor will the changes it induces. Many of these changes raise and will continue to raise the question of the role of the human in this complex and dynamic man–machine interplay. Changes range from the individual level (as a user or as a service provider) up to the macro level with platform innovation leading by and by to monopolistic market structures. Interestingly, research in e-tourism seemed to have moved the other way round: from the macro level and market structures at the beginning to the micro level today. We should not focus (only) on better and faster: we need a long-term perspective. This is the “lesson” of Digital Humanism, looking at the advantages and threats of technology, that it should serve for the better of a society. Let us take seriously Popper and his reference to the responsibility of a scientist (Popper 1969), and ask ourselves: what is the role of IT to rethink and create a socially and ecologically sustainable tourism?

Finally: academia, universities, and research are a place of critical thinking and reflection. This implies the mix of different disciplines as well as technological, organizational, societal, and political perspectives. E-tourism as an interdisciplinary endeavor could provide good practice examples.

## Footnotes

1 Today several of the conference organizers and participants are Professors (some already retired) or successful entrepreneurs.

2 https://sdgs.un.org/goals

3 The Guardian, 12.03.2018.

4 Cassili, A. (2021): Digital Humanism Lecture—What is a 'Truly Ethical' Artificial Intelligence? An end-to-end approach to responsible and humane technological systems. https://www.youtube.com/watch?v=9NWSgny12wY.

5 Or look at the so called “Gigg economy” with the mostly false self-assessment of the independence and freedom of the "producing", but actually mostly self-exploitation.

6 António Guterres on Twitter, Mar 25, 2019; 6:28 PM.

7 There are critics on these classical notions of humanism. For more information have a look at the introduction of (Werthner et al. 2022).

8 https://dighum.ec.tuwien.ac.at/dighum-manifesto/.

9 This material (over 70 h videos) is online accessible at https://dighum.ec.tuwien.ac.at/lectures-program/ and https://www.youtube.com/channel/UC-oCPW9l7IuDvu_J30tqMVw.

10 dighum.ec.tuwien.ac.at/summerschool2022/.

11 I will not distinguish between computer science and informatics, which is not really correct.

12 I do not discuss *Artificial Intelligence*, this would be another paper.

13 An operating system is a system software that manages computer hardware, software resources, and provides common services for computer programs.

14 Elinor Ostrom (Ostrom, 1990), however, showed that there are counter examples where members of a community co-operate or regulate to exploit those resources without collapse. So, not everything is lost.

15 Russel (2021): Digital Humanism Lecture—How not to Destroy the World with AI. https://www.youtube.com/watch?v=apVRH0fbQcQ.

16 There is no good English word for the German “Das Gemeinsame”.

17 https://en.wikipedia.org/wiki/List_of_public_corporations_by_market_capitalization. Although their valuations went down due to the current situation, they are still keeping the leading positions.

18 I define e-tourism as follows: e-tourism denotes the analysis, design, implementation and application of IT / e-commerce solutions in the travel and tourism industry, as well as the analysis (of the impact) of the respective technical/economic processes and market structures. This non-normative definition contains a constructive and an analytical part.

19 Schegg, R.: European Hotel Distribution Study, June 2022. Sample of 3900 hotels in Europe. https://www.hevs.ch/media/document/20/schegg-2022-european-hotel-distribution-survey-hotrec-18may2022-public-3.pdf.

20 i.e., the ratio of output to input, or to use the least amount of inputs to achieve the highest amount of output. There is no place for buffers and other “unnecessary” costly things.

21 Here I refer to the JITT research agenda (Werthner et al. 2015), its classification of open e-tourism research issues provides a framework to identify future developments.

22 Take as an example the car sector, where the established car producers are still struggling to catch up with the software advantage of the new e-car and e-mobility suppliers.

23 Burke (2022): Presentation—Personalization and its Discontents. https://dighum.ec.tuwien.ac.at/dighum-2021-program/”.

24 Horvitz (2021): Digital Humanism Lecture—AI Advances, Responsibilities, and Governance. https://www.youtube.com/watch?v=NXIyYSwq8u0.

25 - > means “this expectation became”. I do not mean that none of the elements of the prediction did not materialize, but negative developments predominate, although these developments are not linear.

26 In 2000 we even had an IFITT workshop on this topic and how it would favor empowerment on bth sides: tourism service providers as well as tourists.

27 But as already mentioned, business seems to continue as usual, with some exceptions. in July 2022 in the two Austrian valleys and ski resorts Pitztal and Ötztal the local population voted in a local referendum (where one could expect that they are in favor since they have economic benefit) against the merger of the two glacier ski areas.

28 Interestingly, the Internet worked well in the pandemic, its basic design principle was redundancy, not optimality; this worked.

29 An exception might be smart tourism, but it is not well defined what “smart” means. One may even say, it means everything which is better then stupid.
